# In Reply:

**DOI:** 10.3325/cmj.2012.53.640

**Published:** 2012-12

**Authors:** Ivan Kruljac, Andreja Marić, Vatroslav Čerina, Hrvoje Ivan Pećina, Petra Šulentić, Milan Vrkljan

We are pleased that our article (1) has provoked interest and encouraged discussion among endocrinologists and pituitary surgeons since it presents data from a rather complex clinical field.

First, we are aware of the “Consensus on Criteria for Cure of Acromegaly,” which define disease remission as suppression of the growth hormone after the glucose load beneath 1 ng/mL and normalization of insulin-like growth factor-1 (IGF-1) 3 to 6 months after surgery (2). However, based on our experience and several other studies (3-5), early postoperative IGF-1 and growth hormone in oral glucose tolerance test is a good predictor of long-term remission. Also, to the best of our knowledge, no studies have shown that early postoperative IGF-1 gave false-positive results. We measured IGF-1 in all patients with acromegaly seven days, three months, and nine months after surgery and performed oral glucose tolerance test in patients with IGF-1 within the upper third of the reference range interval. Measuring IGF-1 in patients with acromegaly seven days after surgery is crucial for early detection of active disease and planning other treatment modalities since residual aggressive somatotropinomas can progress tremendously within three to six months.

Regarding the diagnosis of secondary adrenal insufficiency, neither insulin tolerance test (ITT) nor synacthen test is mandatory to exclude adrenal insufficiency. Performing ITT within six weeks after the surgery has its risks and is not recommended (6). Therefore, we administered hydrocortisone replacement therapy to all our patients postoperatively. In patients receiving hydrocortisone replacement, urinary-free cortisol above 200 nmol/L is a clear sign of over-replacement and compensated pituitary function, while that above 500 nmol/L indicates normal pituitary function (7). Hence, hydrocortisone was gradually decreased in all patients with over-replacement and discontinued if morning cortisol exceeded 500 nmol/L. In others, ITT was performed prior to replacement discontinuation. Patients with urinary-free cortisol from 100 nmol/L to 200 nmol/L on hydrocortisone replacement were considered to have adrenal insufficiency.

Dopamine agonist therapy is the first line treatment for prolactinomas, as highlighted in the introduction to our article. We must point out that surgical patients with microprolactinoma greatly differ from all patients with prolactinoma treated in our Center, and the majority of the patients with microprolactinoma receive dopamine agonist therapy. However, medication therapy is associated with a decrease in life quality, side-effects, and high costs (8). On the other hand, pituitary surgery has substantially improved over the years and the most recent studies report recurrence rates of 5%-10% after surgical treatment of microprolactinomas (cited in our article). Our neurosurgeons have great experience in pituitary surgery (more than 50 operations per year) and therefore we offer our patients endoscopic pituitary surgery as an alternative to dopamine agonist therapy. This approach is in accordance with the practice in most of the leading pituitary centers in the world (9).

In conclusion, treatment of patients with pituitary adenomas is challenging and requires individualized approach in order to achieve good results and low treatment costs. Therefore, the diagnosis and treatment algorithm often differs from clinical guidelines proposed by clinicians and scientists from the world’s wealthiest countries.
